# Comparative phenotypic and genotypic virulence of *Salmonella* strains isolated from Australian layer farms

**DOI:** 10.3389/fmicb.2015.00012

**Published:** 2015-01-23

**Authors:** Andrea R. McWhorter, Kapil K. Chousalkar

**Affiliations:** School of Animal and Veterinary Sciences, University of Adelaide – Roseworthy CampusRoseworthy, SA, Australia

**Keywords:** eggs, *Salmonella*, cell invasion, Caco2, BALB/c mice, *Salmonella* pathogenicity islands

## Abstract

There are over 2500 *Salmonella enterica* serovars that circulate globally. Of these, serovars those classified into subspecies I are the most common cause of human salmonellosis. Many subspecies I *Salmonella* serovars are routinely isolated from egg farm environments but are not frequently associated with causing disease in humans. In this study, virulence profiles were generated for 10 strains of *Salmonella enterica* isolated directly from egg farm environments to investigate their potential public health risk. Three virulence parameters were assessed including *in vitro* invasion, *in vivo* pathogenicity and characterization of genomic variation within five specific pathogenicity islands. These 10 *Salmonella* strains exhibited significant differences in invasion into the human intestinal epithelial cell line, Caco2. Low, moderate, and high invasion patterns were observed and the degree of invasion was dependent on bacterial growth in a nutritive environment. Interestingly, two *Salmonella* strains, *S*. Adelaide and *S*. Bredeney had consistently low invasion. The *S*. Typhimurium definitive types and *S*. Virchow exhibited the greatest cell invasion following growth in Luria Bertani broth. Only the *S*. Typhimurium strains caused disease in BALB/c mice, yet the majority of serovars were consistently detected in feces over the 21 day experiment. Genomic comparison of the five specific pathogenicity islands has shown that variation in virulence is likely multifactorial. Sequence variability was observed primarily in strains with low virulence. In particular, genes involved in forming the structures of the SPI-1 and SPI-2 type 3 secretion systems as well as multiple effector proteins were among the most variable. This variability suggest that serovars with low virulence are likely to have both invasion and within host replication defects that ultimately limit their pathogenicity.

## Introduction

Enteric bacterial pathogens are among the most common causes of diarrheal disease world-wide. Consumption of contaminated food items is frequently the source of pathogens responsible for outbreaks of gastroenteritis. With the globalization of food distribution, contaminated or improperly handled food products have the potential to cause disease in multiple countries. As a consequence, foodborne gastrointestinal diseases could have major socioeconomic impacts in both developed and developing countries (Hendriksen et al., 2011).

*Salmonella* is a diverse group comprised of two major species, *Salmonella (S.) bongori* and *S*. *enterica*. *Salmonella enterica* is further subdivided into six subspecies and is the largest group containing over 2500 serovars (Guibourdenche et al., 2010). *Salmonella* is an intracellular pathogen and depending on both the host species and serovar can cause disease in both humans and animals ranging from mild diarrhea to typhoid fever. Humans generally acquire *Salmonella* through the consumption of contaminated foods, including fruits, vegetables, nuts, dairy, meat, eggs and poultry meat (reviewed in Carrasco et al., 2012). In particular, contaminated raw eggs or improperly handled egg-related products are common sources of *Salmonella* infection (Chen and Jiang, 2014; Threlfall et al., 2014). The incidence of *Salmonella* infection as a consequence of egg or egg product consumption is 23% in the US (Jackson et al., 2013), 39% in Australia (Moffat and Musto, 2013) and has been estimated at 32% in Europe (Pires et al., 2010).

Many *Salmonella* spp. have established a unique niche within poultry environments. The bacteria are able to colonize the gastrointestinal tract of chickens and ultimately spread horizontally and vertically within a flock (reviewed in Foley et al., 2011; Howard et al., 2012). Chicks within the first few days of life are more susceptible than older chickens to *Salmonella* colonization through horizontal transmission of bacteria from a contaminated environment (Foley et al., 2011). Some *Salmonella* serovars such as *S*. Enteritidis can infect chicks through vertical transmission from infected parents (Howard et al., 2012; Sivaramalingam et al., 2013). In addition, infection with *S*. Typhimurium or *S*. Enteritidis can result in a persistent infection or colonization of vital organs in chickens (Wales and Davies, 2011; Gast et al., 2013). Intermittent shedding of *Salmonella* spp. in fecal material can occur as a consequence of physiological and/or environmental stress (Nakamura et al., 1994; Quinteiro et al., 2012; Gole et al., 2014a). Longitudinal epidemiological investigation of *Salmonella* in layer flocks has correlated point-of-lay with peak bacterial loads in feces (Gole et al., 2014a). During these periods, it is likely that egg contamination can occur.

Globally, the two most common *Salmonella* serovars associated with gastrointestinal disease of humans are *S*. Enteriditis and *S*. Typhimurium (Hendriksen et al., 2011). Other non-typhoidal *Salmonella* (NTS) serovars are also responsible for causing considerable disease but they do not have widespread global distribution and their prevalence is location dependent (Hendriksen et al., 2011). Previous virulence studies utilizing primarily European or North American serovars have described considerable variation in their *in vitro* invasive capacity (Suez et al., 2013) as well as their ability to cause disease in mouse models (Swearingen et al., 2012). In Australia, NTS particularly *S*. Typhimurium definitive types are also frequently responsible for egg product related food poisoning outbreaks (Ozfoodnet Working Group, 2012) but there is currently limited characterization of their virulence.

Comparative genomic analyses of *Salmonella enterica* has revealed that there is considerable variation in virulence elements across the species as a whole (Jacobsen et al., 2011). The genome of *S*. Enteriditis, for example, possesses “regions of difference” (ROD) or clusters of coding sequences not present in all serovars, in particular *S*. Typhimurium (Thomson et al., 2008). Several of these RODs including two coding sequences within SPI-19 (SEN1001 and SEN1002), a cluster of genes within ROD21 linked to tRNA-asnT (SEN1970 to SEN1999), the *peg* fimbrial operon (SEN2144A to SEN2145B) as well as genes within ROD40 that are components of a type I restriction modification system (SEN4290 to SEN4292) are responsible for conferring increased *in vivo* virulence to *S*. Enteriditis (Silva et al., 2012). Characterization of phenotypic virulence and genotypic variability across multiple NTS serovars has, however, not been fully explored. In the present study, we have selected *Salmonella* serovars that are commonly isolated from layer hen environments that are also associated with human salmonellosis as well as other serovars whose incidence of disease is low (in Australia). Although it is likely that these strains share virulence mechanisms, our aim is to generate a virulence profile and identify genetic differences that lead to variation in overall pathogenicity.

For this study, 10 NTS strains isolated directly from various point sources (e.g., dust, feces, litter) in a layer hen environment were selected. *Salmonella* Typhimurium definitive types (DT) 44, DT135, DT170, DT193 and Virchow are frequently isolated from contaminated egg products during human salmonellosis outbreaks in Australia while the others are less commonly associated with disease (South Australian *Salmonella* Reference Laboratory, 2010, 2011, 2012, 2013). The invasive ability of NTS strains was investigated using both an *in vitro* human intestinal epithelial cell model as well as an *in vivo* mouse model. Whole genome sequencing of the selected 10 strains was also performed and the sequences of five specific pathogenicity islands with roles in both *in vitro* invasion and infection *in vivo* were analyzed and compared with the published reference strain *S*. Typhimurium LT2. LT2 was selected as a reference strain because the mechanisms of its *in vitro* and *in vivo* pathogenicity have been widely studied and the entire annotated genome is publicly available from the National Center for Biotechnology Information (NCBI).

## Materials and methods

### Bacterial strains

*Salmonella enterica* strains *S*. Adelaide, *S*. Bredeney, *S*. Cerro, *S*. Orion, *S*. Senftenberg, *S*. Virchow, and *S*. Typhimurium definitive types 44 (DT 44), 170=108 (DT170=108), 135 (DT135), and 193 (DT193) were selected for this study. All *Salmonella* strains were originally isolated from chicken fecal samples, dust or litter and were obtained from the *Salmonella* Reference Laboratory (Adelaide, South Australia). Table 1 lists the serovars selected for this study, the source and the number of times over a 4 year period that they were isolated from South Australian egg farms. Individual strains were stored long term at −80°C. Bacteria were recovered from freezing by streaking onto nutrient agar plates and incubated overnight at 37°C. Bacterial suspensions for experiments were prepared using either 0.9% saline or Luria Bertani broth (10 g tryptone, 5 g yeast extract, 10 NaCl per 1 L).

**Table 1:** **The frequency of annual detection of the 10 strains selected for this study**.

**Collection number**	**Serovar**	**Isolation source**	**Number of times *Salmonella* serovars were isolated from egg farms**			
			**2010**	**2011**	**2012**	**2013**
KC14ADL	Adelaide	Feces	0	2	0	7
KC14BRD	Bredeney	Feces	0	1	0	0
KC14CER	Cerro	Feces/ Litter	2	0	0	0
KC14ORI	Orion	Dust	1	3	1	0
KC14SEF	Senftenberg	Feces	3	5	0	0
KC14VIR	Virchow	Feces	12	12	1	1
KC14TY44	Typhimurium DT44	Feces	43	35	12	1
KC14TY170_108	Typhimurium DT170=108	Feces	7	16	2	13
KC14TY135	Typhimurium DT135	Feces	48	79	17	9
KC14TY193	Typhimurium DT193	Feces/ Litter	2	14	0	7

*Strains were isolated from either egg shell wash or Australian layer farm environment (South Australian *Salmonella* Reference Laboratory, 2010, 2011, 2012, 2013)*.

### Cell culture

The human intestinal epithelial cell line, Caco2 (ATCC HTB-37), was selected for the gentamicin protection invasion assay. Cells were cultured in Dulbecco's Modified Eagle media (DMEM) (HyClone, Australia) containing 4 mM glutamine, glucose, 10% (vol/vol) fetal bovine serum (Hyclone, Australia), and 100 U/ml penicillin and 100 μg/ml streptomycin (ThermoScientific, Australia) at 37°C with 5% CO_2_. Cells were used between passages 5 and 10.

### *In vitro* bacterial invasion assay

The invasive capacity of each *Salmonella* strain selected for this study was characterized using the gentamicin protection assay on polarized Caco2 cells. Briefly, Caco2 cells were first expanded in growth media (DMEM containing 10% FBS and 100 U/ml penicillin and 100 μg/ml streptomycin). Cells were then sub-cultured and placed into wells of a 48 well tissue culture tray (NUNC) at a concentration of 10^4^ cells per well. A polarized cell monolayer was obtained by maintaining the culture in growth media and monitored for the production of alkaline phosphatase using a SensoLyte pNPP detection kit following manufacturer instructions (AnaSpec, USA). Once alkaline phosphatase production stabilized for 48 h (generally after 13–15 days), invasion experiments were conducted. Tissue culture media was changed every 48 h during polarization.

The gentamicin minimum inhibitory concentration (MIC) was determined for all strains included in this study using the Clinical and Laboratory Standard Institute (CLSI) guidelines (Clinical and Laboratory Standards Institues, 2013). The MIC for all strains was less than 0.25 μg/ml gentamycin. Prior to invasion experiments, bacteria were recovered from freezing by plating on to nutrient agar plates and incubating at 37°C overnight. Bacterial suspensions were created by suspending individual colonies in normal saline to an OD600 between 0.15 and 0.20 (corresponding to 10^8^ bacteria cells/ml). Individual *Salmonella* strains were added separately to wells of the tissue culture tray to a multiplicity of infection (MOI) of 100 in DMEM containing no supplements. Prior to the addition of bacteria, the polarized Caco2 monolayer was washed three times with DMEM containing no supplements. Bacteria were incubated with the cell monolayer for 2 h and then removed by aspiration. Caco2 cells were then washed two times with DMEM containing 400 μg/ml gentamicin as per (Mickael et al., 2010) and incubated at 37°C for 15 min. The gentamicin was removed and the cell monolayers were washed three times with DMEM. Cells were lysed in 10% Triton X for 30 min at 37°C. The cell lysate was collected and serial 10-fold dilutions were prepared. Dilutions were plated onto Xylose lysine deoxycholate agar (XLD) (Oxoid, Australia) plates and incubated at 37°C overnight. Bacterial colonies were enumerated. Data are represented at mean percent recovery. Experiments were performed with duplicate replications and were repeated five times.

Additional invasion experiments were performed using bacteria grown to stationary phase in LB. Twenty-four hours prior to the invasion assay, a single colony was taken from each plate and placed into separate tubes containing 3 ml of LB broth. Tubes were incubated with shaking (100 rpm) for 6 h at 37°C. After 6 h, 10 μl of the starter culture was added to tubes containing 5 ml of LB. Bacteria were incubated with shaking overnight at 37°C. Suspensions were diluted to an OD600 between 0.15 and 0.2. Invasion assays proceeded as described above and were repeated five times.

### *In vivo* pathogenicity

Inbred female BALB/c mice were obtained from Laboratory Animal Services (Adelaide, South Australia). All mice were between 6 and 9 weeks of age at the time of infection and were maintained under specific pathogen free conditions prior to and during experiments. All experiments were performed with the approval of the University of Adelaide Ethics Committee and in accordance with the guidelines of the National Health and Medical Research Council.

Inocula were prepared by growing bacteria to stationary phase in LB broth. Inoculum concentration was confirmed by plating serial 10-fold dilutions. Mice were inoculated with either 10^3^ or 10^5^ CFU bacteria by oral gavage. Following infection, mice were monitored for signs of infection. Scores were given to disease parameters such as coat ruffling, change in behavior, hunching, dehydration, consumption of food, and percent loss of mass. Changes in coat appearance or posture had a scoring range of zero to three; zero indicating that the animal was normal and three that a severe effect was observed. Scores were also given for behavior, evidence of dehydration, feed intake and the presence of tremors. Scores for these clinical parameters ranged from zero to two, zero for normal behavior and two for a severe behavior. If an animal exhibited a clinical score of 5 at any stage during the experiment it was humanely euthanized. Clinical scoring was performed by a single individual for consistency and objectivity.

### Collection of fecal samples and culture methods

Fecal pellets were collected from each mouse at day 3, 6, 9, 12, 15, and 18 days post infection (p.i.) and processed for *Salmonella* isolation by culture method described previously (Gole et al., 2014a). Briefly, 100 mg of fecal material was incubated in 1 ml of buffered peptone water (1:10) and incubated at 37°C overnight. 100 μl of this sample was added to Rappaport-Vassiliadis Soya peptone broth (RVS, Oxoid, Australia) and incubated at 42°C overnight. A 100 μl of the RVS culture was then spread onto XLD agar plates (Oxoid, Australia) and a Brilliance *Salmonella* agar (Oxoid, Australia).

#### DNA extraction from fecal samples

Fecal samples were collected from 0 to 18 days p.i. from each treatment group. DNA from feces (0.2 g) was extracted using a QIAamp DNA stool mini kit (Qiagen, Australia) according to manufacturer instructions. Extracted DNA was quantified using a Nanodrop ND1000 (ThermoScientific, Australia) and stored at −80°C until used for real-time (RT)-PCR. Five nanograms of fecal DNA were used for the RT-PCR reaction.

#### Q-PCR (real time PCR)

*Salmonella* shedding in fecal material was quantified using real time PCR (RT-PCR). RT-PCR was performed using a Rotor Gene 3000 real time PCR machine (Qiagen, Australia) and a TaqMan® *Salmonella enterica* detection Kit (Applied Biosystems, Australia). Each reaction contained 9 μl of qPCR supermix and 6 μl of DNA template (5 ng) in a total reaction volume of 15 μl. The cycling parameters were 95°C for 10 min, then 40 cycles at 95°C for 15 s followed by 60°C for 60 s. All real time PCR runs included a negative and positive control. The data was analyzed by Two-Way analysis of variance (ANOVA).

A standard curve was generated by preparing a serial dilution of the *Salmonella* Typhimurium DT135 strain used in this study. Bacteria were resuscitated on nutrient agar overnight at 37°C. The individual isolated colonies were then suspended in 2 mL of phosphate buffered saline (PBS) and matched with a 0.5 McFarland standard (bioMerieux Australia). Serial dilutions were performed to achieve 10^8^ CFU/ml. The CFUs were confirmed by spreading serial dilutions on XLD agar plates. In order to determine, the limit of detection of Q-PCR, fecal samples were spiked with various concentrations (10^8^–10^0^ CFU/mL) of *Salmonella* Typhimurium DT135. qPCR was performed on serial dilutions (10^8^–10^0^) of genomic DNA and a proportionality relationship was produced by plotting the Ct value against the logarithm CFU number. *Salmonella* copies were calculated using a standard curve prepared by serial 10 fold dilution of a cultured *Salmonella* spp. During each reaction, internal standards comprised of serial dilutions of genomic DNA of *Salmonella* Typhimurium DT135 were included. A negative control was also included in each reaction.

### Whole genome sequencing

Whole genome sequencing was performed on the 10 *Salmonella* strains selected for this study. *Salmonella* were cultured on nutrient agar. A single colony was selected and grown overnight at 37°C with shaking (100 rpm) in brain heart infusion broth. Bacterial DNA was purified from the overnight culture using the Promega Wizard Genomic DNA purification Kit (Promega, USA). Quality of DNA was assessed by Nanodrop ND1000 (ThermoScientific, Australia) and agarose gel. Sequencing was performed by the Australian Genomic Research Facility using the Illumina MiSeq platform.

#### Sequence analysis

Sequences were analyzed using CLC Genomics Workbench (version 7.0.4). Sequences were trimmed and de novo assembly was performed to obtain large contigs no less than 1000 base pairs. Single gene analysis of five *Salmonella* specific pathogenicity islands (SPI) 1, 2, 3, 4, and 5 was performed using *Salmonella* Typhimurium LT2 (NC_003197) as a reference strain. Sequences have been uploaded to NCBI and accession numbers are listed in Table 2.

**Table 2:** **NCBI accession numbers for pathogenicity island sequences**.

	**SPI-1**	**SPI-2**	**SPI-3**	**SPI-4**	**SPI-5**
Adelaide	KP279305	KP258186	KP279315	KP234065	KP234055
Bredeney	KP279306	KP258187	KP279316	KP234066	KP234056
Cerro	KP279307	KP258188	KP279317	KP234067	KP234057
Orion	KP279308	KP258189	KP279318	KP234068	KP234058
Senftenberg	KP279309	KP258190	KP279319	KP234069	KP234059
Typhimurium DT44	KP279310	KP258191	KP279320	KP234070	KP234060
Typhimurium DT170=108	KP279311	KP258192	KP279321	KP234071	KP234061
Typhimurium DT135	KP279312	KP258193	KP279322	KP234072	KP234062
Typhimurium DT193	KP279313	KP258194	KP279323	KP234073	KP234063
Virchow	KP279314	KP258195	KP279324	KP234074	KP234064

### Statistical analysis

Kruskal-Wallis ANOVA with post hoc analysis utilizing Dunn's multiple comparisons test was used to determine statistical significance of invasive capacity of *Salmonella* strains in Caco2 cells. All statistical analyses were performed using GraphPad Prism version 6.0. *P*-values less than 0.05 were considered statistically significant.

## Results

### Comparative invasive capacity of multiple *Salmonella* strains

**Figure 1:**
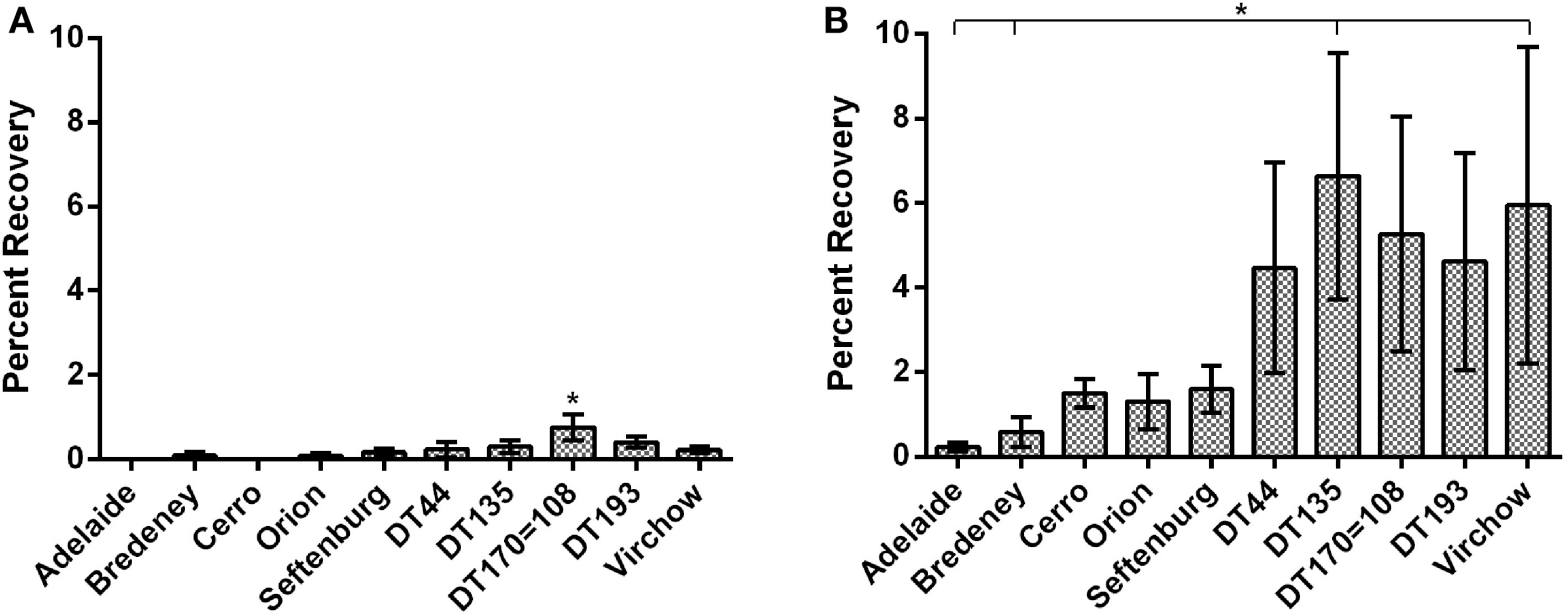
**The invasive potential of *Salmonella* strains was characterized using polarized Caco2 cells**. Data are represented as percent invasion. **(A)** Invasion potential of bacteria suspended in normal saline. *S*. Typhimurium DT170=108 suspended in normal saline was significantly more invasive than all other strains (*p* < 0.05). **(B)** Growth to stationary phage in LB substantially increased the invasive capacity of most strains. A significant effect of serovar was detected for bacteria cultured in LB (*p* < 0.01). *S*. Typhimurium DT44, DT135, DT170=108 and *S*. Virchow exhibited the highest mean percent invasion.

The invasive capacity of the 10 NTS strains was first investigated using bacteria suspended in normal saline. Data are presented as mean percent recovery of individual *Salmonella* strains in relation to the original inoculum (Figure 1A). Overall cell invasion was limited for all strains, with mean percent recoveries ranging from 0.03 to 0.75. There were, however, significant differences detected amongst *Salmonella* strains tested. *S*. Typhimurium DT 170=108 exhibited the greatest invasive capacity (mean percent recovery, 0.75 ± 0.16) and was found to be significantly more invasive compared to all other strains tested (*p* < 0.05) (Figure 1A). *S*. Typhimurium DT DT44, DT135, DT193, and *S*. Virchow exhibited moderate invasion while *S*. Bredeney, *S*. Orion and *S*. Senftenberg all exhibited low invasive capacity. *S*. Adelaide and *S*. Cerro exhibited negligible invasion (Figure 1A).

*In vitro* growth media substantially affects the expression of genes required for *in vitro* invasion (Mills and Finlay, 1994; Ibarra et al., 2010). Therefore, the gentamicin invasion protection experiment was repeated with bacteria cultured to stationary phase in Luria Bertani (LB) broth. A significant increase in invasive capacity (*p* < 0.001) was observed for all strains grown in LB broth as compared with suspensions in physiological saline. Increase in invasive capacity ranged from 6.9 to 375 fold, with *S*. Cerro exhibiting the greatest change. The *S*. Typhimurium definitive types DT44, DT135, DT170=108, DT193 as well as *S*. Virchow exhibited the greatest overall invasive potential. DT135 and *S*. Virchow had the highest mean percent recoveries, 6.6 ± 1.5 and 5.9 ± 1.8 respectively, and were significantly more invasive than either *S*. Adelaide or *S*. Bredeney (*p* < 0.05) (Figure 1B). Interestingly, despite growth in LB both *S*. Adelaide and *S*. Bredeney retained low invasion capacities (Figure 1B).

### Comparative *in vivo* pathogenicity of *Salmonella* strains

To further investigate the pathogenicity of the selected *Salmonella* strains, groups of seven BALB/c mice were inoculated with either 10^3^ or 10^5^ colony forming unit (CFU) of individual strains. These doses were selected as they represent a range of bacterial contamination detected on the surface of an egg shell (Gole et al., 2014b).

**Figure 2:**
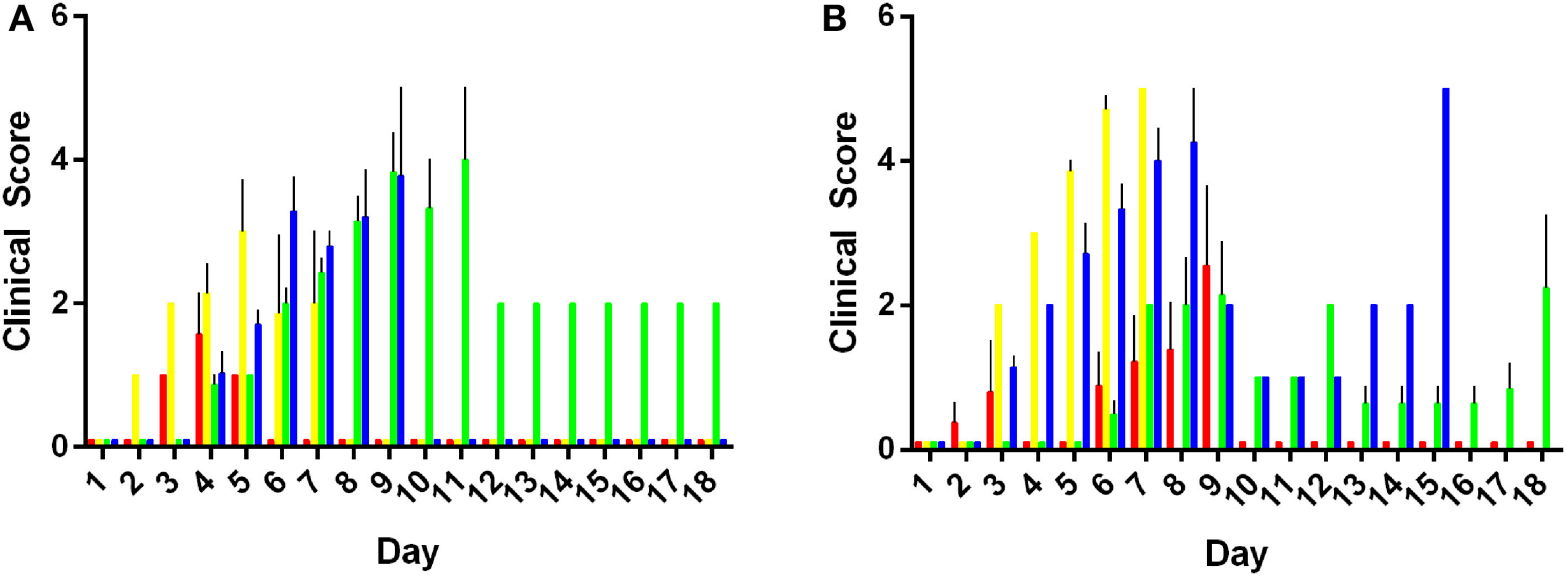
**Morbidity in mice infected with *Salmonella* Typhimurium definitive types**. Clinical signs of infection appeared from day 2 with 10^3^ CFU of individual serovars **(A)**. Peak morbidity in low dose animals ranged between day 6 and 10 post infection. Morbidity was observed in animals inoculated with 10^5^ CFU from day and peaked between days 5 and 9 post infection (p.i.) **(B)**. DT44 (red), DT135 (yellow), DT170=108 (green) and DT193 (blue).

Morbidity of mice was characterized using a clinical scoring system. Data obtained for the morbidity scores are presented as mean clinical score ranging from zero to five taken for an entire experimental group. Results are summarized in Figure 2. Animals inoculated with 10^3^ or 10^5^ CFU of *S*. Adelaide, *S*. Bredeney, *S*. Cerro, *S*. Orion, and *S*. Senftenberg did not exhibit any clinical signs of infection over the course of the entire 21 day experiment. As such, their clinical scores were zero and not included in the morbidity analysis. Mice inoculated with both doses of *S*. Virchow exhibited very mild clinical symptoms during the first 72 h of the experiment. Clinical scores for animals infected with *S*. Virchow did not rise above one during this period (data not shown).

Mice inoculated with either 10^3^ or 10^5^ CFU of *S*. Typhimurium DT44, DT135, DT170=108, or DT193 exhibited the greatest overall degree of morbidity (Figure 2). Mice inoculated with 10^3^ CFU of the *S*. Typhimurium strains exhibited a range of clinical symptoms over the course of the experiment. In the low dose group, DT44 caused the least morbidity, very mild clinical symptoms from day 3 to 5 were observed but by day 6 post-infection (p.i.) all animals had recovered. The greatest morbidity in the 10^3^ CFU group was observed in both DT135 and DT193. Hunching behavior, coat ruffling and weight loss were observed from day 2 p.i. for animals infected with 10^3^ CFU of DT135. Morbidity for this group increased rapidly from day 3 to day 5 and by day 7 p.i. all animals were euthanized. Similarly, animals infected with DT193 exhibited morbidity from day 5 p.i. which increased till day 7 p.i.; by day 9 p.i., all animals were euthanized. Animals infected with DT170=108 exhibited clinical symptoms from day 4 p.i. Morbidity in this group peaked between days 7 and 9 p.i.. Only one animal remained in this group till the end of experiment at day 21 p.i..

As in the low dose group, clinical symptoms were first observed at day 3 p.i. in mice inoculated with 10^5^ CFU of the *S*. Typhimurium strains. The degree of morbidity however was substantially increased in mice receiving the high dose of bacteria. Four mice in the DT44 group exhibited severe morbidity between days 6–9 but the other three animals did not display any clinical signs of infection over the course of the experiment. Animals inoculated with 10^5^ CFU of both DT135 and DT193 exhibited the greatest amount of morbidity over the course of the experiment. For both of these strains, clinical symptoms were observed from day 3 and morbidity scores peaked at day 7 and 8 respectively. The majority of animals in the DT170=108 group exhibited minor morbidity over the course of the experiment.

**Figure 3:**
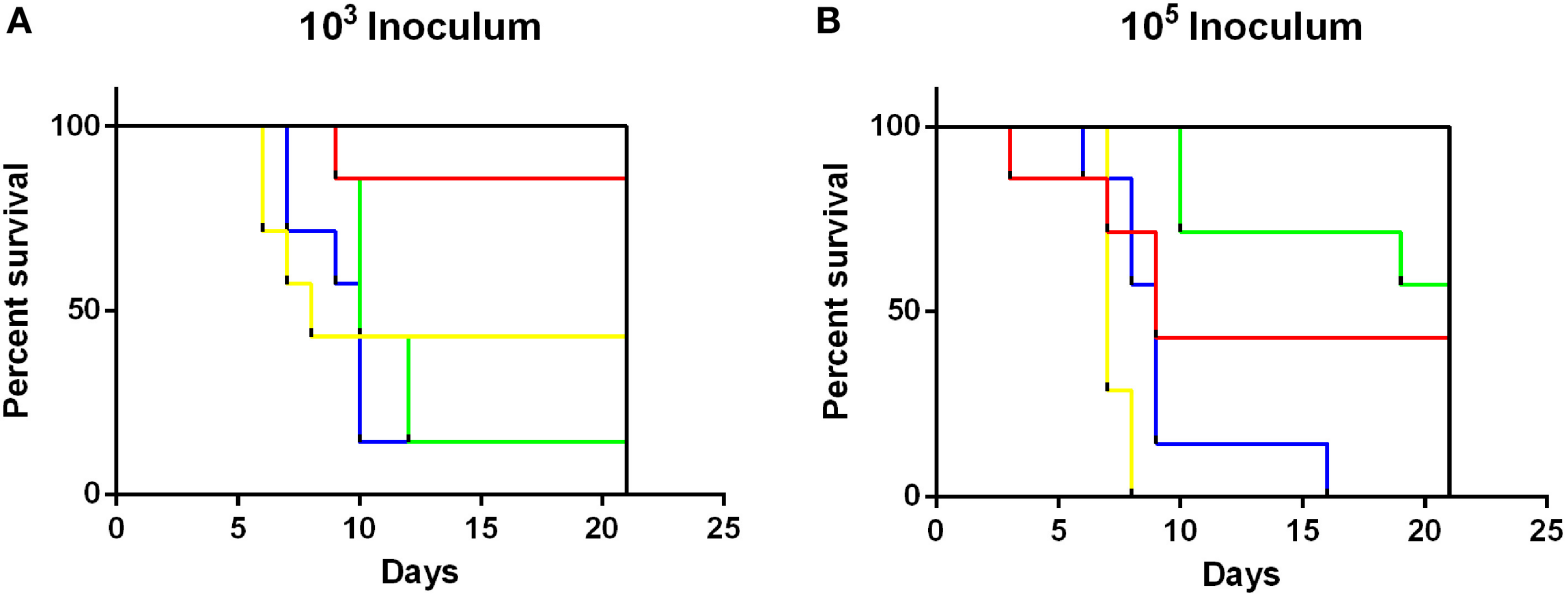
**Survival curves for mice inoculated with either 10^3^ or 10^5^ CFU of *S*. Typhimurium definitive types**. *S*. Typhimurium definitive types DT44 (red), DT135 (yellow), DT170=08 (green) and DT193 (blue) exhibited significantly greater mortality at both the 10^3^ **(A)** and 10^5^ **(B)** dose, than all other strains (black line) tested in this study. Mice inoculated with 10^3^ CFU of either DT135, DT170=108 or DT193 all had significantly greater mortality than DT44 (*p* < 0.01). The greatest mortality was observed for mice inoculated with 10^5^ CFU of DT135.

Survivability of NTS infection was also assessed (Figure 3). Throughout the experiment, no mortalities were recorded in animals infected with either 10^3^ or 10^5^ CFU of *S*. Adelaide, *S*. Bredeney, *S*. Cerro, *S*. Orion, *S*. Senftenberg, or *S*. Virchow. Survival of mice infected with all *S*. Typhimurium definitive types had significantly different survival curves (Mantel-Cox log rank test, *p* < 0.001) than all other strains at both doses. Interestingly, the mortality of DT170=108 at the 10^3^ dose was higher than 10^5^ dose. Significant differences were detected in survivability between *S*. Typhimurium groups (*p* < 0.01). At the low dose DT135, DT170=108, and DT193 were significantly more likely to cause mortality than DT44 (*p* < 0.01). At the 10^5^ dose DT135 was significantly more virulent than all other *S*. Typhimurium definitive types included in this study (*p* < 0.01) (Figure 3).

### Fecal shedding of *Salmonella* strains

The shedding of *Salmonella* in feces is an important mechanism of transmission of the bacteria from host to host. In this study, *Salmonella* shedding was monitored both by culture isolation as well as by a qPCR method. The TaqMan *Salmonella enterica* PCR assay does not enable the quantification of positive fecal samples. Therefore, a standard curve generated by preparing a serial 10-fold dilution of a known concentration of *Salmonella* spp. (10^8^–10^0^ CFU) was used. The standard curve produced a slope of −3.2, a y intercept of 39.4 and R^2^ of 0.91. A cut-off Ct of 33.7 was used to exclude detection of false positives. A Ct of 33.7 corresponded to 50 CFU of *Salmonella*. Amplification was not recorded in the negative control (LB) samples or any of the treatment groups at day 0 of infection. Shedding of *Salmonella* into the feces was consistently observed by both culture and qPCR methods during the course of the experiment for all strains at both doses (Table 3). For *S*. Typhimurium DT135, *S*. Orion, *S*. Virchow and *S*. Bredeney treatment groups, a significant difference in *Salmonella* shedding between the dose and days p.i. was detected (*p* < 0.0001). In addition, significant interaction between dose and days p.i. was observed (*p* < 0.0001). A significant effect between serovar and day p.i. was observed for *S*. Typhimurium DT44, DT193 (*p* < 0.0005), DT108=170, *S*. Adelaide, *S*. Senftenberg and *S*. Cerro (*p* < 0.0001). A significant interaction between day p.i. and dose was observed for all groups except for DT44, DT 193, and *S*. Cerro. The highest amount of *Salmonella* detected in feces was detected in mice inoculated with 10^3^ CFU *S*. Senftenberg with a mean of 1.3 × 10^8^ ± 1.3 × 10^8^ CFU/g fecal material.

**Table 3:**
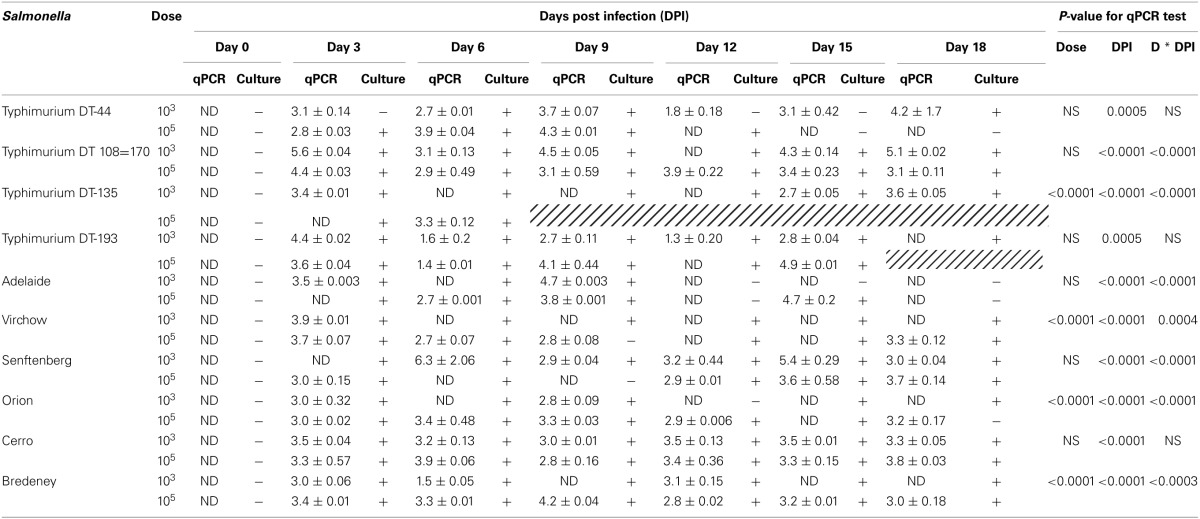
**Real time qPCR and culture detection of *Salmonella* in fecal samples collected from BALB/c mice inoculated with 10^3^ and 10^3^CFU**.

*Amplification was not recorded in negative control samples or any of the treatment groups at day 0 of infection. ND, limit of detection for qPCR assay. (+) Indicates culture positive samples, while (−) indicates culture negative. Cells filled with hashed lines indicate no animals remained in that group*.

### Comparative genomics of SPIs of multiple non-typhoidal *Salmonella* strains

To determine whether genotypic variability contributed to pathogenic verses non-pathogenic phenotypes observed in this study, whole genome sequencing was performed on each of the 10 *Salmonella* strains. Single gene analysis of specific pathogenicity islands (SPI) 1, 2, 3, 4, and 5 was performed. Amino acid sequences for each gene were generated *in silico* and compared with the corresponding sequence of *S*. Typhimurium LT2.

SPI-1 contains 39 genes involved in the formation of a Type III transmembrane secretion system (T3SS) as well as multiple effector proteins. The greatest sequence variation was observed in *avrA, srpB, orgC, prgI, sptP, and sipA* for *S*. Adelaide, *S*. Bredeney, *S*. Cerro, *S*. Orion, and *S*. Senftenberg (Table 4). *S*. Adelaide and *S*. Orion both lacked the *avrA* gene. For *S*. Bredeney, a single base pair deletion shifts the open reading frame causing a premature stop codon at 292. Base pair substitution in the *avrA* coding sequence of *S*. Virchow causes a premature stop at amino acid 251. *S*. Typhimurium DT44 possesses a triple base pair deletion in *avrA* that truncates the protein by one amino acid but does not affect the open reading frame.

**Table 4:**
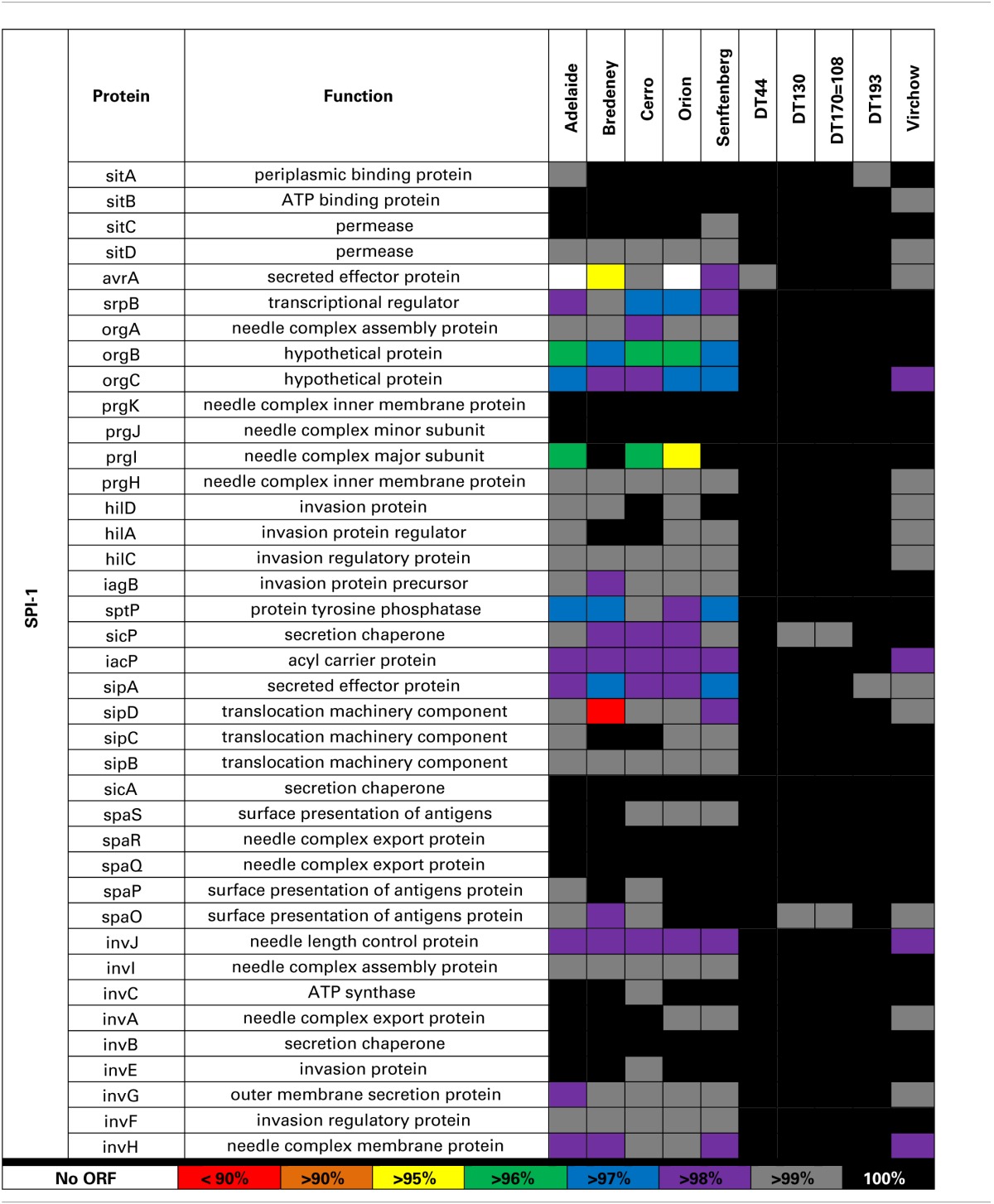
**Comparative analysis of SPI-1 sequences from 10 NTS strains**.

*The amino acid sequence of SPI-1 of each of the 10 Salmonella strains included in this study was compared with the reference strain LT2. Genes are color coded according to their amino acid similarity to the reference strain LT2. Sections denoted with white indicate that no open reading frame (ORF) was present*.

Amino acid variability was also observed for orgB, orgC and prgI in all strains except the four Typhimurium definitive types. An insertion of 10 base pairs at the 3′ end of the orgB sequence was detected in *S*. Adelaide, *S*. Bredeney, *S*. Cerro, *S*. Orion, and *S*. Senftenberg. This leads to a premature stop at amino acid 224 disrupting the open reading frame. Considerable amino acid variability was also observed for prgI in *S*. Adelaide, *S*. Cerro and *S*. Orion. *S*. Bredeney had a nine base pair deletion in *sipD* causing a three amino acid truncation of the protein. Minor amino acid variability was observed for the sptP sequence of *S*. Adelaide, *S*. Bredeney, and *S*. Senftenberg. A single base pair substitution was detected in the *sptP* sequence of *S*. Virchow creating a premature stop at position 128 of a 544 amino acid protein.

A second T3SS is encoded by SPI-2 has 31 genes in multiple operons. Sequence variability amongst the 10 *Salmonella* strains examined in this study was found in the *ssa* and *sse* operons in *S*. Adelaide, *S*. Bredeney, *S*. Cerro, *S*. Orion, *S*. Senftenberg, and *S*. Virchow (Table 5).

**Table 5:**
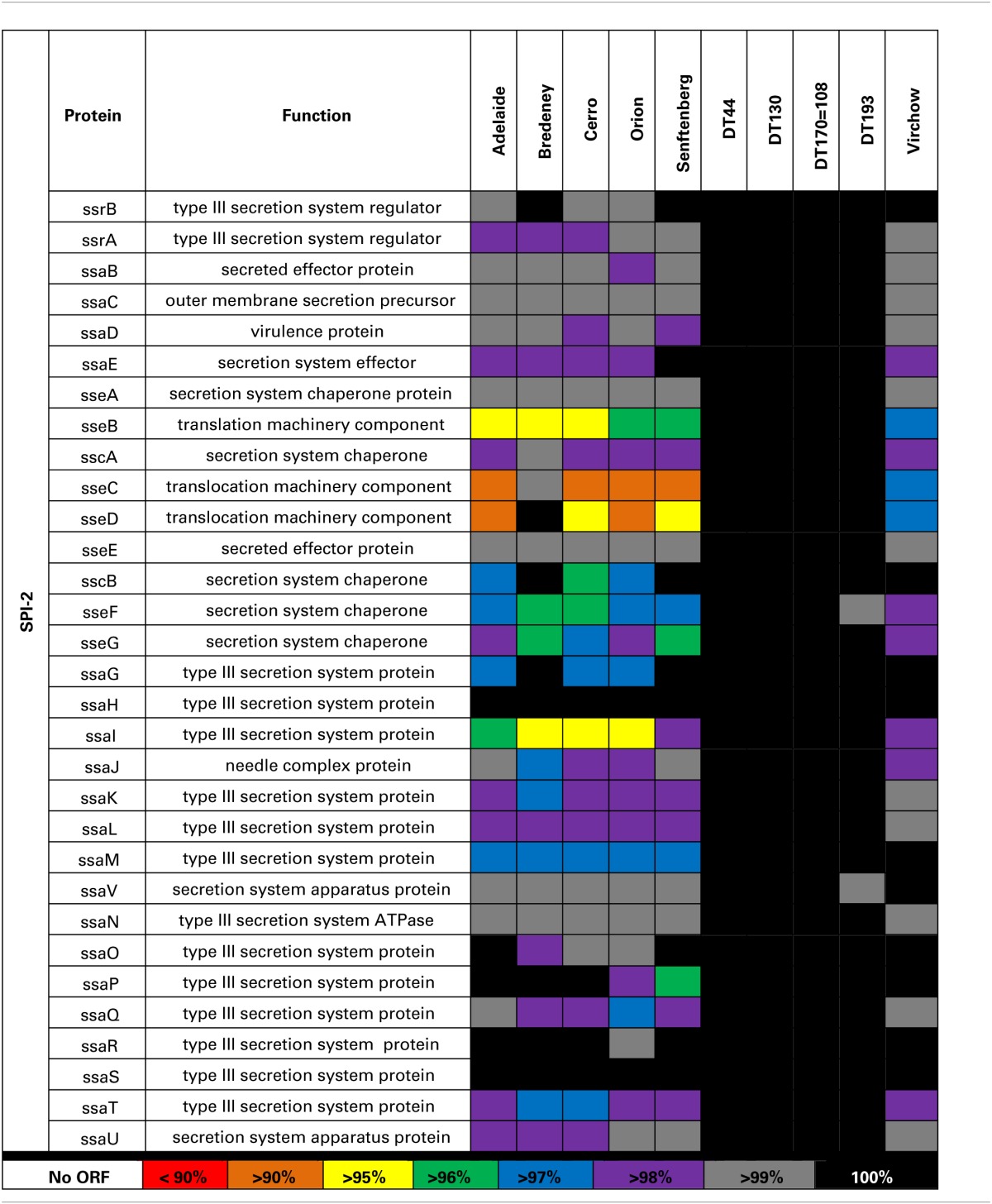
**Sequence variability within SPI-2**.

*Genes are color coded according to their amino acid similarity to the reference strain LT2. Sections denoted with white indicate that no open reading frame (ORF) was present. Considerable amino acid variability was observed in coding sequences for proteins that make up the type 3 secretion system*.

Analysis of SPI-3 genes revealed that, 69.2% of the strains analyzed in this study, had greater than 98% amino acid homology with the LT2 strain. Sequence variation was primarily observed in the region between STM3752 and rhuM. *S*. Adelaide, *S*. Cerro and *S*. Senftenberg were found to lack multiple genes within the STM372-rhuM region. STM3754 was absent in *S*. Adelaide, *S*. Cerro, and *S*. Senftenberg (Table 6). *S*. Senftenberg lacked the gene *sugR*. *S*. Virchow and *S*. Bredeney lacked the entire region (Table 6).

**Table 6:**
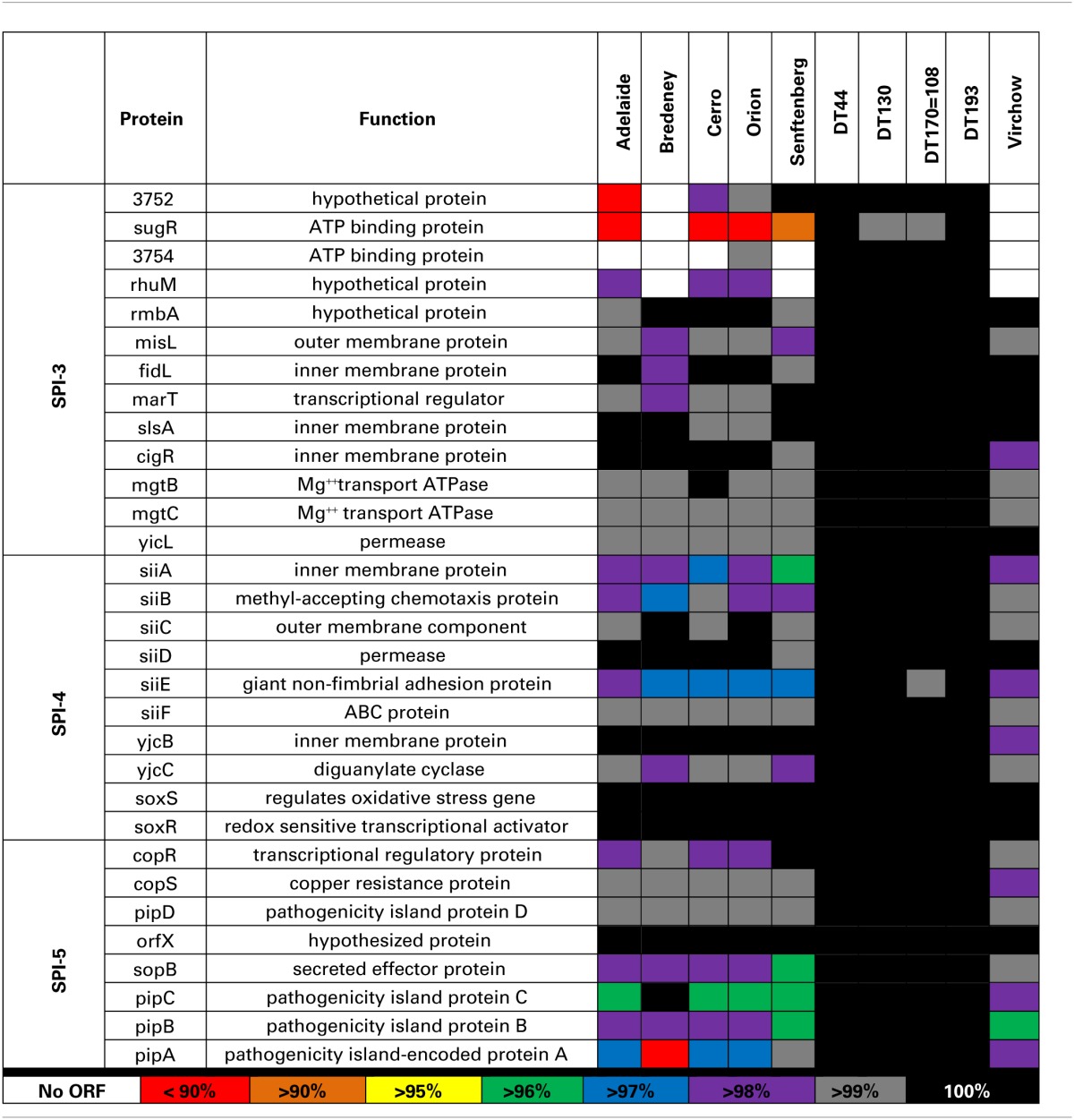
**Amino acid variability of SPI-3, -4 and -5**.

*Genes are color coded according to their amino acid similarity to the reference strain LT2. Sections denoted with white indicate that no open reading frame (ORF) was present. The SPI-3 region between STM3752 and rhuM as well as siiA, B and E in SPI-4 was among the most variable. In SPI-5, the pip operon exhibited the highest level of amino acid variation*.

SPI-4 contains a single operon, *siiABCDE* (McClelland et al., 2001). In general, all 10 *Salmonella* strains were highly conserved for SPI-4; 70% of the genes exhibited greater than 98% homology to LT2 (Table 6). The highest amino acid variability was observed in siiA, B and E for *S*. Bredeney, *S*. Cerro, *S*. Orion, and *S*. Senftenberg.

The pathogenicity island, SPI-5, encodes six genes involved in enteropathogenesis (Wood et al., 1998). Genes within SPI-5 code for effector proteins translocated by the T3SSs of both SPI-1 and SPI-2 (Knodler et al., 2002). Sequence variability was observed primarily in the *pipABC* operon (Table 6). A large deletion in *S*. Bredeney pipA alters the open reading frame. Considerable amino acid variation was also observed in the pipA sequence of *S*. Adelaide, *S*. Cerro, and *S*. Orion. Minor amino acid variability was also observed for pipC and sopB in *S*. Adelaide, *S*. Cerro, *S*. Orion, and *S*. Senftenberg.

## Discussion

Surface contamination of an egg shell with *Salmonella* is an important contributing factor to outbreaks of human salmonellosis. Higher loads of bacteria on the egg shell can contribute to contaminated hands in a kitchen environment as well as contaminated utensils (Humphrey et al., 1994). Viable bacteria can be isolated from an egg shell up to 21 days post-lay depending on storage conditions (Messens et al., 2006). Furthermore, *S*. Enteriditis, *S*. Typhimurium, and *S*. Heidelberg present in chicken feces are able penetrate into the interior of eggs and subsequently multiply in storage (reviewed in Chen and Jiang, 2014). The *in vitro* and *in vivo* experiments described here were designed to determine whether *Salmonella* strains represent an equal risk to public health.

The *in vitro* invasion results highlight clear differences in the invasive capacity of the 10 selected *Salmonella* strains. These invasion data also indicate that some *Salmonella* strains may have constitutive expression of genes enabling them to invade even at low levels under non-nutritive conditions, ultimately providing them with a competitive advantage. Pathogenic bacteria are able to sense their environment and respond through the up regulation of genes that facilitate their within-host survival (Ellermeier and Slauch, 2007). Furthermore, it has been reported that *in vitro* growth media stimulates the expression of genes required for *Salmonella* invasion in cultured epithelial cells (Mills and Finlay, 1994; Ibarra et al., 2010). As such, it is not surprising that substantial increases in invasion were observed amongst the selected strains post-enrichment.

Current *in vivo* pathogenicity data are largely limited to *Salmonella* serovars that most commonly cause disease. It has recently been shown that not all NTS serovars possess the same *in vivo* virulence (Swearingen et al., 2012). The BALB/c mouse strain was selected for this study as it has been previously been shown to be susceptible to infection with *Salmonella* (Plant and Glynn, 1976). The selection of a susceptible mouse strain was warranted as human cases of salmonellosis occur most commonly amongst the young, elderly or immunocompromised (Jones et al., 2008b). *S*. Adelaide, *S*. Bredeney, *S*. Cerro, *S*. Orion, and *S*. Senftenberg exhibited limited or moderate invasive capacity during our *in vitro* studies hence their lack of *in vivo* virulence was not unexpected. *S*. Virchow is consistently associated with cases of human salmonellosis yet the strain used in this study lacked the ability to cause disease in mice. This result was somewhat surprising as the *S*. Virchow strain was among the most invasive in our *in vitro* invasion experiments. This discrepancy between cell invasiveness and *in vivo* virulence has also been described for strains of *S*. Abortusovis and *S*. Montevideo (Swearingen et al., 2012) as well as *S*. Enteriditis (Shah et al., 2011) but the mechanisms responsible for this outcome were not investigated.

The pathogenicity of *S*. Typhimurium has been studied extensively over the past decade as a model for systemic typhoid fever. As a consequence, the virulence mechanisms that *S*. Typhimurium employs during *in vivo* infection have been well characterized (reviewed in Fabrega and Vila, 2013). In this study, we found that mice inoculated with either 10^3^ or 10^5^ CFU of *S*. Typhimurium DT44, DT135, DT170=108, or DT193 exhibited the greatest overall degree of morbidity, yet there were significant differences between their disease capacity. These findings were consistent with our *in-vitro* cell invasive data. A multitude of factors including fimbriae, virulence plasmids and the normal expression of virulence genes likely contribute to this variation in virulence and has recently been demonstrated for *S*. Enteriditis (Shah et al., 2011). The pathogenicity of *S*. Typhimurium has been studied extensively over the past decade as a model for systemic typhoid fever. As a consequence, the virulence mechanisms that *S*. Typhimurium employs during *in vivo* infection have been well characterized (reviewed in Fabrega and Vila, 2013). There is, however, limited evidence characterizing variation in *in vivo* virulence amongst different Australian *S*. Typhimurium definitive types. Interestingly, mice infected with 10^3^ CFU of DT170=108 strain exhibited greater mortality than those inoculated with 10^5^ CFU. It has been shown that *Salmonella* can be more pathogenic at lower doses by evading CD4^+^ T cell activation (Srinivasan et al., 2004). In this study, increased mortality was, however, observed only for DT170=108. Thus, it could be hypothesized that such a dose dependent impact on T cell responsiveness could vary between serotypes.

Host to host transmission of *Salmonella* occurs predominantly via the oral-fecal route. As a consequence, the duration of shedding post-infection as well as the total bacterial load within feces of an infected individual can contribute to the transmission of disease. It is interesting to note that despite a lack of evidence of disease, fecal shedding was also observed over the duration of this study for *S*. Bredeney, *S*. Cerro, *S*. Orion, *S*. Senftenberg, and *S*. Virchow. *S*. Bredeney and *S*. Cerro were detected using both *Salmonella* detection methods at both doses over the course of the entire experiment. These results are consistent with other virulence studies of NTS serovars demonstrating that avirulent strains are capable of establishing persistent intestinal infection that results in the consistent shedding of bacteria (Swearingen et al., 2012). Mice infected with 10^3^ CFU of *S*. Adelaide, however, stopped shedding bacteria after day 9 p.i.. Some disparity between culture and qPCR results was observed, in particular for mice infected with DT44. It should be noted that PCR methods detect both viable and non-viable bacteria within a sample. A double enrichment protocol was used to culture *Salmonella* from the mouse fecal samples. Thus, it is likely that the bacteria detected by qPCR in the 10^3^ DT44 treatment groups were non-viable.

The genotypic variability observed within the five *Salmonella* pathogenicity islands for the 10 strains included in this study was largely limited to *S*. Adelaide, *S*. Bredeney, *S*. Cerro, *S*. Orion, *S*. Senftenberg and to a lesser extent *S*. Virchow. SPIs are highly conserved across *Salmonella enterica* yet it is variations within these genes that have the potential to affect virulence. Genes within these pathogenicity islands enable *Salmonella* to invade host cells, replicate and evade the immune response (reviewed in Fabrega and Vila, 2013). Genomic variability within these pathogenicity islands may contribute to the wide range of virulence observed for members of *S. enterica*. Sequence analysis of SPI1, 2, 3, 4, and 5 was performed to determine if genotypic variability contributed to the observed range of pathogenicity. Amino acid variation was largely limited to *S*. Adelaide, *S*. Bredeney, *S*. Cerro, *S*. Orion, *S*. Senftenberg and to a lesser extent *S*. Virchow.

In SPI-1, amino acid variation was observed in avrA, srpB, orgC, prgI, sptP, and sipA. *AvrA* encodes 33 kDa protein that is translocated into host intestinal epithelial cells during infection (Hardt and Galán, 1997) and is important for modulating the host immune response (Wu et al., 2012) as well as intracellular survival of the bacterium (Jones et al., 2008a; Wu et al., 2012). In mice, infection with an *avrA* deletion mutant has been shown to increase disease (Jones et al., 2008a; Wu et al., 2012). No evidence of aggravated disease was observed for *S*. Adelaide or *S*. Orion.

The *prg, org, inv* and *spa* operons encode the needle complex of the T3SS while the *sic* and *sip* operons encode effector proteins (Fabrega and Vila, 2013). OrgB and orgA are part of the effector sorting platform of the SPI-1 T3SS (Kawamoto et al., 2013). It is unclear whether the premature stop observed for *S*. Adelaide, *S*. Bredeney, *S*. Cerro, *S*. Orion, and *S*. Senftenberg would have any functional effect. The needle complex is formed by 120 copies of prgI which interacts with sipD to form the pore structure of the T3SS that embeds in host cell membranes (Rathinavelan et al., 2014). The amino acid sequence variability observed for prgI may affect how the needle complex in these strains is formed. The formation of the pore structure may also be affected if sipD is not able to interact normally with prgI.

*sptP* encodes a protein tyrosine phosphatase that is translocated across the SPI-1 T3SS and is involved in the initial disruption of the actin cytoskeleton (Fu and Galán, 1998) as well as the modulation of the host immune response (Choi et al., 2013). Minor amino acid variability was observed for several strains but without further functional studies, it is unclear whether the variability observed in sptP would have functional effects on the protein.

A second T3SS is encoded by SPI-2 (T3SS-2) is involved in the translocation of effector proteins across the *Salmonella*-containing vacuole (Figueira and Holden, 2012). The greatest sequence variability was observed in the *ssa* and *sse* operons. Genes within the *ssa* operon encode a portion of the T3SS-2 that forms within the membrane of the bacteria. Proteins encoded by ssaG and ssaI form part of the needle complex that extends beyond the *Salmonella* outer membrane (Kuhle and Hensel, 2004). Mutant *Salmonella* lacking either *ssaG* or *ssaI* have previously been shown to be unable to replicate intracellularly or translocate effector proteins through T3SS-2 (Chakravortty et al., 2005). While the strains included in this study possessed a complete open reading frame, it is possible that the amino acid variability observed could prevent normal formation of the needle complex. The sseB, C and D proteins of SPI-2 are secreted onto the surface of the bacterium and are required for the translocation of effectors (Nikolaus et al., 2001; Ruiz-Albert et al., 2003; Fabrega and Vila, 2013). SseC and sseD form the pore structure across the vacuole membrane (Kuhle and Hensel, 2004). Mutations in *sseC* and *sseD* have a significant impact on the virulence in mice (Klein and Jones, 2001). Serovars that have mutations in these genes were severely attenuated and unable to secrete effectors through the T3SS (reviewed in Ruiz-Albert et al., 2003). SseB forms part of the translocon through the vacuole membrane. Mutants of *sseB* were able to replicate within host cells but unable to escape, thus limiting their dispersal (Grant et al., 2012).

SPI-3 has 10 open reading frames that encode virulence determinants with highly diverse functions. Sequence variation was largely observed in the region between STM3752 and *rhuM*. Sequence diversity has also been demonstrated for this region across *S. enterica* by several groups (reviewed in Fabrega and Vila, 2013).

SPI-4 is a 27 KB region within the *Salmonella* genome (Wong et al., 1998). During infection, SPI-4 acts in consort with SPI-1 to initiate invasion in to host epithelial cells (Gerlach et al., 2008). Deletion of SPI-4 attenuated the virulence of both *S*. Typhimurium and *S*. Enteriditis in mice (Kiss et al., 2007). siiA, B and E exhibited the greatest variability in amino acid sequence for *S*. Bredeney, *S*. Cerro, *S*. Orion, and *S*. Senftenberg. The gene *siiE* encodes a giant, non-fimbrial adhesion protein that enables the bacterium to adhere the apical surface of a host cell a process that is required for SPI-1 T3SS mediated invasion (Gerlach et al., 2007, 2008; Main-Hester et al., 2008). It has recently been shown that siiA and siiB form a proton channel within the inner membrane of the bacteria and that they are regulatory proteins for siiE (Wille et al., 2014). The amino acid variability observed in the siiA and siiB sequences of the *Salmonella* strains included in this study may have an effect on the regulation of *siiE* in these strains.

SPI-5 encodes six genes that play a role in the enteropathogenesis of *Salmonella* spp. (Wood et al., 1998). The highest variability was observed in the *pipABC* operon. pipA is an effector protein that is translocated across the *Salmonella* vacuole by the SPI-2 T3SS and is important for the development of systemic disease in mice (Knodler et al., 2002). The pipC protein functions as a chaperone and is also involved in the stabilization of SopB (Darwin et al., 2001).

At this stage, the relationship between genotypic variability and reduced cellular invasion is not clear and requires further functional study. It is also important to note that there are additional pathogenicity islands encoded within the *Salmonella* genome that are not included in this study. Genes from these SPIs may also contribute to functional variation. Furthermore, we have not discussed the potential contribution of the variation in fimbriae and flagella genes.

The experiments described here were targeted at identifying whether different *Salmonella* strains represent an equal risk to public health. We have shown that there are clear differences in the overall virulence capacity of the 10 selected strains and that the mechanisms driving this variation are multifactorial and appear to be serovar dependent. A caveat is, however, that only a single strain from each serovar was included in this study. It is highly probable that the differences observed within the SPIs are specific for the strains selected for this study. Within serovar analyses are necessary to define the overall pathogenic potential of that serovar.

## Author contributions

Andrea R. McWhorter and Kapil K. Chousalkar conceived and designed the experiments. Andrea R. McWhorter performed all invasion assays and sequence analysis. The *in vivo* infection trial was performed by both Andrea R. McWhorter and Kapil K. Chousalkar. Quantitative-PCR was performed by Kapil K. Chousalkar. Both authors contributed equally to statistical analysis and manuscript preparation.

### Conflict of interest statement

Neither author has a conflict of interest.
